# Exosomal circ_DLGAP4 promotes diabetic kidney disease progression by sponging miR-143 and targeting ERBB3/NF-κB/MMP-2 axis

**DOI:** 10.1038/s41419-020-03169-3

**Published:** 2020-11-23

**Authors:** Shoujun Bai, Xiaoyan Xiong, Bo Tang, Tingting Ji, Xiaoying Li, Xiaolei Qu, Weiliang Li

**Affiliations:** 1 Department of Nephrology, Qingpu Branch of Zhongshan Hospital Affiliated to Fudan University, 1158 Gongyuan East Road, Qingpu District, 201700 Shanghai, People’s Republic of China; 2 Department of Urology, Qingpu Branch of Zhongshan Hospital Affiliated to Fudan University, 1158 Gongyuan East Road, Qingpu District, 201700 Shanghai, People’s Republic of China

**Keywords:** Diagnostic markers, Diabetic nephropathy

## Abstract

Diabetic kidney disease (DKD) is closely associated with the high risk of cardiovascular disease and mortality. Exosomal circRNAs can exert significant roles in the pathology of various diseases. Nevertheless, the role of exosomal circRNAs in DKD progression remains barely known. Circular RNA DLGAP4 has been reported to be in involved in acute ischemic stroke. In our study, we found exosomal circ_DLGAP4 was increased in the exosomes isolated from HG-treated mesangial cells (MCs), DKD patients, and DKD rat models compared with the corresponding normal subjects. Then, we observed that exo-circ_DLGAP4 significantly promoted proliferation and fibrosis of MCs cells. Moreover, to study the underlying mechanism of circ_DLGAP4 in regulating DKD, bioinformatics method was consulted and miR-143 was predicted as its target. The direct correlation between miR-143 and circ_DLGAP4 was validated in MCs. MCs proliferation and fibrosis were increased by circ_DLGAP4, which could be decreased by mimic-miR-143. Next, elevated expression of Erb-b2 receptor tyrosine kinase 3 (ERBB3) is involved in various diseases. However, the function of ERBB3 in DKD development remains poorly known. Next, ERBB3 was predicted as the downstream target for miR-143. It was displayed that circ_DLGAP4 promoted proliferation and fibrosis of MCs by sponging miR-143 and regulating ERBB3/NF-κB/MMP-2 axis. Meanwhile, the loss of exo-circ_DLGAP4 induced miR-143 and repressed ERBB3/NF-κB/MMP-2 expression in MCs. Subsequently, in vivo assays were performed and it was proved that overexpression of circ_DLGAP4 markedly promoted DKD progression in vivo via modulating miR-143/ERBB3/NF-κB/MMP-2. In conclusion, we indicated that exosomal circ_DLGAP4 could prove a novel insight for DKD development.

## Introduction

Diabetes mellitus is a common metabolic disorders worldwide and it can contribute to almost 3.2 million deaths each year^1,2^. Meanwhile, diabetic kidney diseases (DKD) is cardiovascular event and it is a frequent cause of end-stage renal disease^3,4^. Due to its increasing prevalence and excessive risk of cardiovascular mortality, DKD is becoming a big burden of global public health^5^. During the past years, although the first-line therapy is able to slow, but it cannot prevent DKD development. It is significant to identify innovative therapeutic strategies for DKD^6^.

Exosomes are vesicle-like bodies and they are secreted into the extracellular space. Exosomes function a lot in transmitting proteins, lipids, and nucleic acids. The concept of exosomes were first proposed when studying normal cells and tumor cells^7^. As a new type of biological marker, exosomes act as crucial carriers of signal transmission in many diseases, including DKD^8–11^. For example, exosomes from HG-treated glomerular endothelial cells can induce the EMT phenotypes and podocytes dysfunction^12^. Urinary exo-miR-451-5p can be identified as an early biomarker of DKD rats^13^. In addition, exosome from adipose-derived stem cells can reduce DKD progression via inducing autophagy flux and repressing apoptosis in podocytes^14^.

CircRNAs are noncoding RNAs with closed loop, and they are lack of 5′ and 3′ polarity and a polyadenylated tail^15^. Recently, increasing studies have elucidated circRNAs have various functions, especially sponging microRNAs^16,17^. Moreover, the roles of circRNAs have been reported in many diseases, including kidney diseases^18,19^. CircRNA DLGAP4 is a novel circRNA and it originates from the exons 8–10 of DLGAP4, and DLGAP4 is reported to be related to functions of neurons and brain diseases^20,21^. However, the function of circ-DLGAP4 in DKD progression remains poorly known.

Currently, our work investigated the expression of circ-DLGAP4 and its correlation with DKD. We found exosomal circ_DLGAP4 could induce mesangial cells (MCs) cell proliferation and fibrosis in DKD progression by sponging miR-143 and modulating Erb-b2 receptor tyrosine kinase 3 (ERBB3)/NF-κB/MMP-2.

## Results

### Exo-circ_DLGAP4 was elevated in DKD

**Fig. 1:**
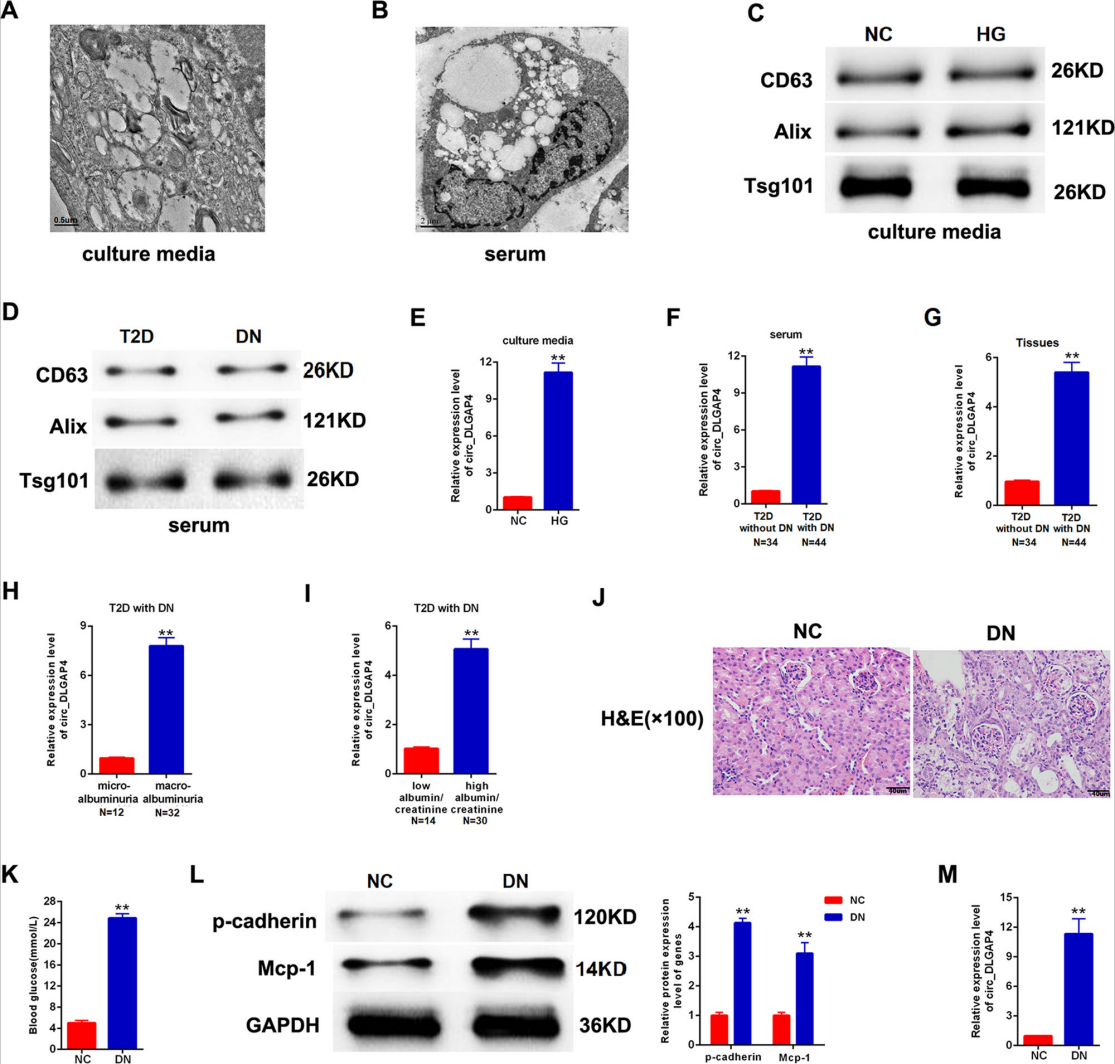
The expression patterns of exo-circ_DLGAP4 in DKD. **A** Scanning of exosomes isolated from culture media of MCs using electron microscopy. **B** Scanning of exosomes isolated from human serum using electron microscopy. **C** CD63, Alix, and Tsg101 protein expression in culture media of MCs was detected using western blotting analysis. **D** CD63, Alix, and Tsg101 protein expression in human serum was evaluated using western blotting analysis. **E** The expression level of circ_DLGAP4 was detected in exosomes isolated from culture media of MCs using qRT-PCR. MCs were treated with NG (5.5 mM) or HG (25 mM) for 24 h. **F** The expression level of circ_DLGAP4 was tested in exosomes isolated from human serum samples of T2D and DKD patients. **G** The expression level of circ_DLGAP4 was detected in human serum samples of T2D patients without DKD (*n* = 34) or with DKD (*n* = 44). **H** Expression levels of circ_DLGAP4 was detected in DKD with microalbuminuria (*n* = 12) or with macroalbuminuria (*n* = 32). **I** The expression level of circ_DLGAP4 were detected in DKD with low eGFR (*n* = 14) or high eGFR (*n* = 30). **J** HE staining in NC and DKD rat model. *n* = 8 in each group. **K** Blood glucose was detected in NC and DKD rat model. **L** Western blotting analysis of p-cadherin and Mcp-1 in NC and DKD rat model. **M** The expression level of circ_DLGAP4 was detected in NC and DKD rat model. Three independent experiments were conducted. Error bars stand for the mean ± SD of at least triplicate experiments; **P < 0.01.

CircRNA has been widely reported in exosomes, few studies have concentrated on the profile of exosomal circRNAs in DKD. Firstly, we isolated exosomes from culture media of MCs and human serum samples. Then, these exosomes were analyzed using electron microscopy and demonstrated in Fig. 1A, B. The exosomal markers CD63, Alix, and Tsg101 protein expression were clearly tested in exosomes from both MCs culture media and human serum samples evaluated, using western blotting analysis (Fig. 1C,D). Circ_DLGAP4 in exosomes of HG-treated MCs, DKD patients, and T2D with DKD patients showed an increased level compared with that in normal subjects, as shown in Figs. 1E–G. Next, circ_DLGAP4 expression was increased in DKD with macroalbuminuria and high eGFR (Fig. 1H, I). DKD rat models were established, and renal tubular epithelial cell edema and glomerular mesangial hyperplasia were observed in DKD rats, as shown using hematoxylin–eosin (HE) staining in Fig. 1J. Blood glucose and p-cadherin and Mcp-1 protein expression were greatly induced in DKD rat model (Fig. 1K, L). Finally, the expression level of circ_DLGAP4 was also elevated in DKD rat model compared to the NC group in Fig. 1M.

### Exo-circ_DLGAP4 promoted growth and fibrosis of MCs cells

**Fig. 2:**
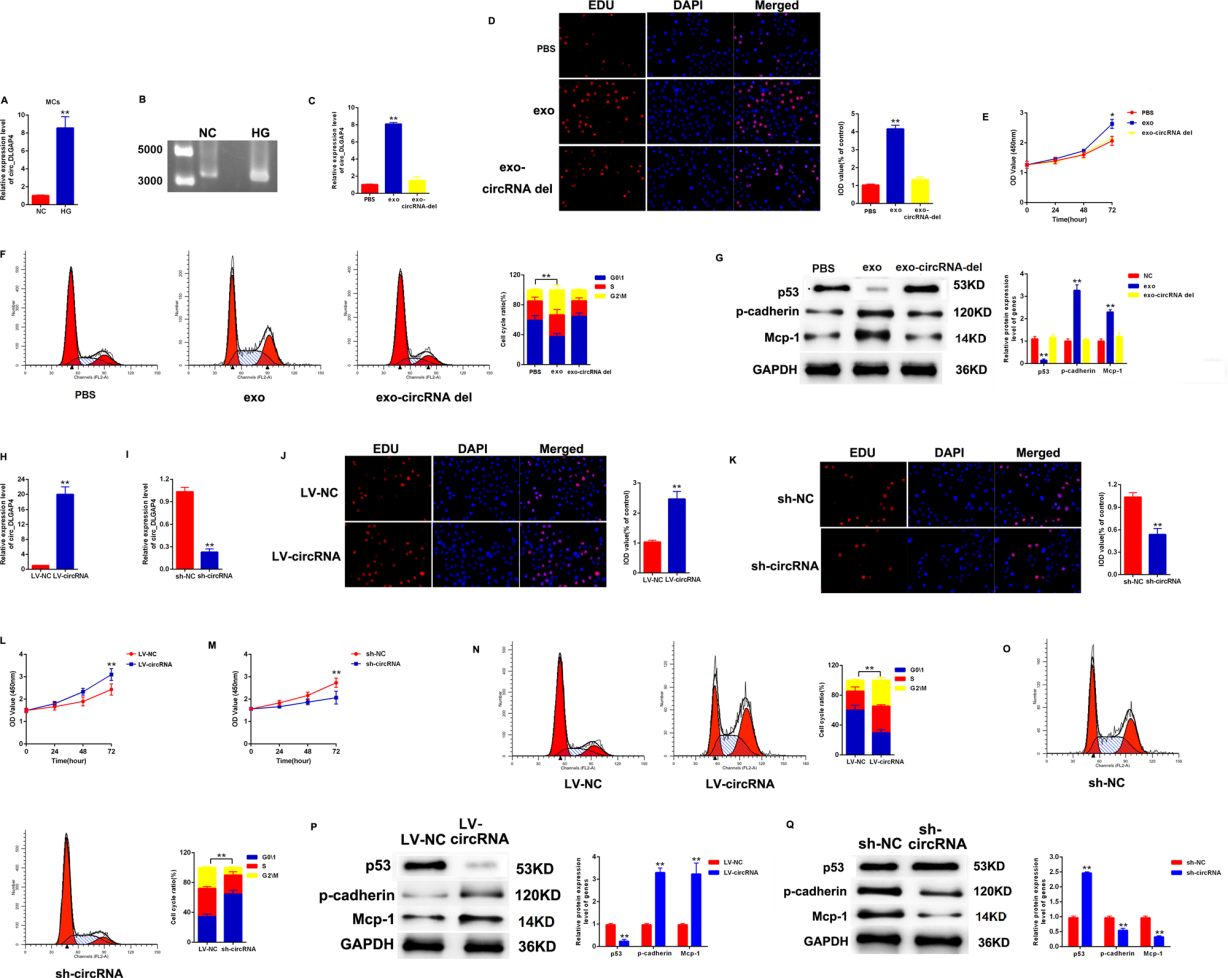
Exo-circ_DLGAP4 promoted growth and fibrosis of MCs. **A** The expression level of circ_DLGAP4 was detected in exosomes isolated from MCs culture media treated with NC or HG. **B** Validation of circ_DLGAP4 using reverse transcription PCR (RT-PCR) analysis. **C** The expression level of circ_DLGAP4 was detected in PBS, exosomes, and exosomes-circRNA del. Exosomes were collected after si-circRNA transfection for 48 h in MCs in exosomes-circRNA del group. **D**, **E** EdU and CCK-8 were carried out to test MCs proliferation and viability. **F** Flow cytometric assay was used to evaluate cell cycle progression in MCs. **G** Western blotting was used to test p-53, p-cadherin, and Mcp-1 protein expression. **H**, **I** Efficiency of LV-circ_DLGAP4 and sh-circ_DLGAP4 in MCs were detected by qPCR. **J**, **K** EdU assay was carried out to test cell proliferation. **L**, **M** Results of CCK-8 assays. **N**, **O** Cell cycle analysis using flow cytometric assay. **P**, **Q** Western blotting assay was used to assess p-53, p-cadherin, and Mcp-1 protein expression in MCs. Three independent experiments were conducted. Error bars stand for the mean ± SD of at least triplicate experiments; *P < 0.05, **P < 0.01.

Then, we studied the effect of exo-circ_DLGAP4 on MCs growth and fibrosis. We found that the expression level of circ_DLGAP4 in exosomes isolated from MCs culture media was increased by HG in Fig. 2A. Then, in Fig. 2B, validation of circ_DLGAP4 was carried out using reverse transcription PCR (RT-PCR) analysis. Moreover, the expression level of circ_DLGAP4 in exosomes isolated from the culture media of MCs under NG was inhibited by circ_DLGAP4 siRNA, as exhibited in Fig. 2C. Then, EdU and CCK-8 were carried out to test MCs proliferation and we found that exo-circ_DLGAP4 induced cell proliferation, while siRNA of circ_DLGAP4 reduced cell proliferation greatly in Fig. 2D, E. In Fig. 2F, flow cytometric assay indicated that cell cycle progression was blocked in G2/phase by exo-circ_DLGAP4. Western blotting was used to test DKD markers, including p-53, p-cadherin, and Mcp-1 protein expression. p-53, p-cadherin, and Mcp-1 protein levels were significantly increased by exo-circ_DLGAP4, while reduced after deletion of exo-circ_DLGAP4, as exhibited in Fig. 2G. In addition, MCs were infected with LV-circ_DLGAP4 or sh-circ_DLGAP4. The efficiency of LV-circ_DLGAP4 and sh-circ_DLGAP4 was confirmed in Fig. 2H, I. EdU assay and CCK-8 assay proved that MCs proliferation was upregulated by circ_DLGAP4 overexpression, while downregulated by loss of circ_DLGAP4, as indicated in Fig. 2J–M. Cell cycle was arrested in G2/M by LV-circ_DLGAP4, while sh-circ_DLGAP4 blocked cell cycle in G0/G1 phase in Fig. 2N, O. Fibrosis marker of MCs, including p-cadherin and Mcp-1 protein expression and p-53 level were induced by LV-circ_DLGAP4, while reduced by sh-circ_DLGAP4 in MCs in Fig. 2P, Q.

### miR-143 was a direct target of circ_DLGAP4

**Fig. 3:**
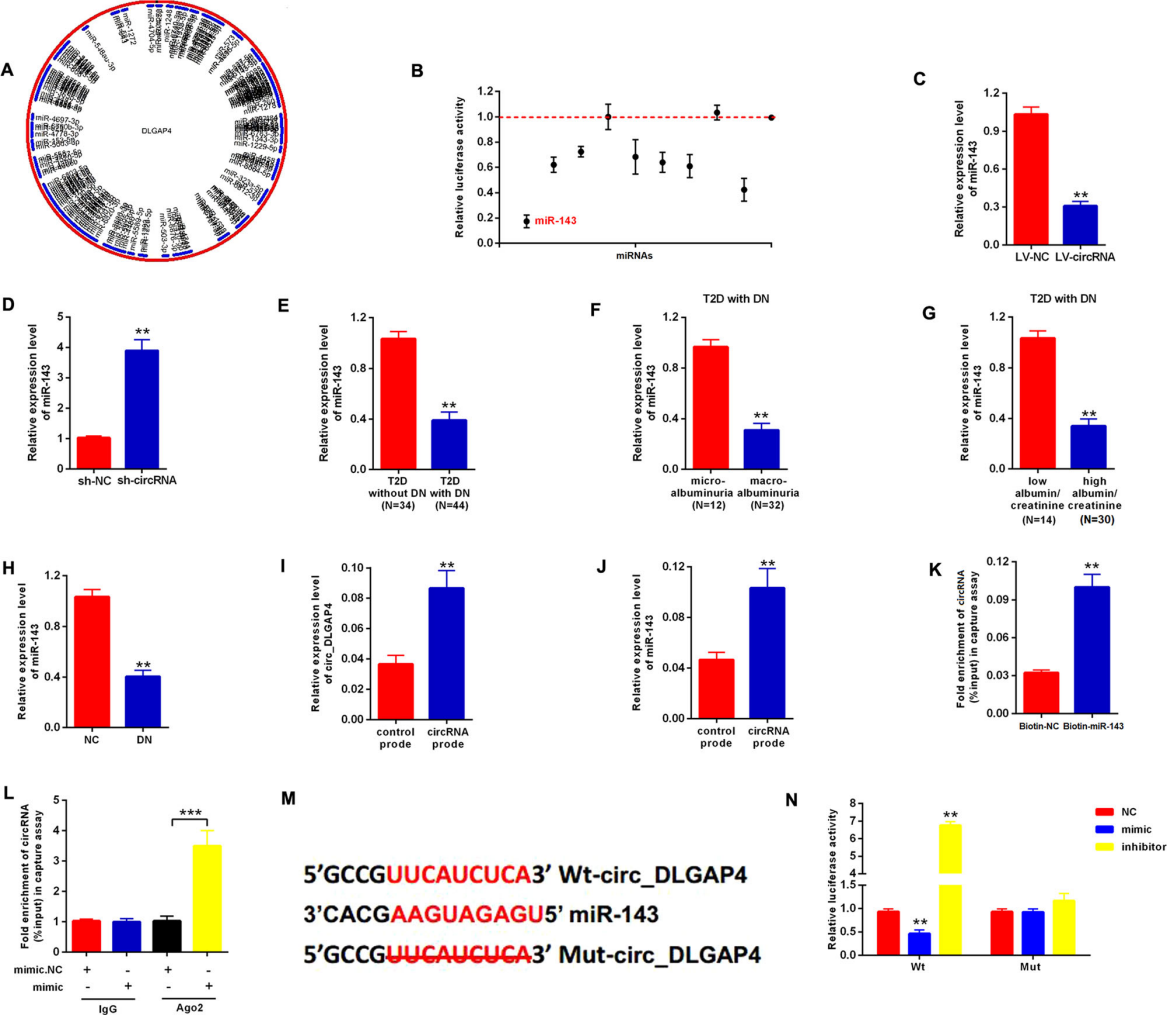
miR-143 was the direct target of circ_DLGAP4. **A** The schematic model indicated the putative binding sites of predicted miRNAs on circ_DLGAP4 (http://www.bioinf.com.cn/). **B** Luciferase activity of circ_DLGAP4 in MCs transfected with miRNA mimics with putative binding to the circ_DLGAP4 sequence. **C**, **D** The expression level of miR-143 in MCs transfected with LV-circ_DLGAP4 or sh-circ_DLGAP4. **E** The expression level of miR-143 in T2D patients without DKD (*n* = 34) or with DKD (*n* = 44). **F** Expression levels of miR-143 was detected in DKD with microalbuminuria (*n* = 12) or with macroalbuminuria (*n* = 32). **G** The expression level of miR-143 were detected in DKD with low eGFR (*n* = 14) or high eGFR (*n* = 30). **H** The expression level of miR-143 was decreased in rat DKD model. **I** circ_DLGAP4 in cell lysis was pulled down and enriched with circ_DLGAP4 specific probe in MCs. **J** miR-143 was pulled down and enriched with circ_DLGAP4-specific probe in MCs. **K** Biotin-coupled miR-143 captured a fold change of circ_DLGAP4 in the complex as compared with biotin-coupled NC in biotin-coupled miRNA capture. **L** RIP was performed using Ago2 antibody in MCs cells transfected with miR-143 mimic or mimic NC. The enrichment of circ_DLGAP4 was detected. **M** The direct binding sites between circ_DLGAP4 and miR-143 were presented. **N** Luciferase reporter activity of circ_DLGAP4 in MCs cells co-transfected with miR-143 mimic, inhibitor, or NC. Three independent experiments were conducted. Error bars stand for the mean ± SD of at least triplicate experiments; **P < 0.01, ***P < 0.001.

Moreover, to find out the underlying mechanism of circ_DLGAP4 in regulating DKD, we consulted bioinformatics method using http://www.bioinf.com.cn/ and a schematic model indicated the putative binding sites of predicted miRNAs on circ_DLGAP4, as displayed in Fig. 3A. Top ten miRNAs were listed in Supplementary Table 1. Then, in Fig. 3B, luciferase activity of circ_DLGAP4 in MCs transfected with ten miRNA mimics with putative binding to the circ_DLGAP4 sequence were detected. miR-143 mimics exhibited the lowest luciferase activity. The expression level of miR-143 in MCs transfected with LV-circ_DLGAP4 or sh-circ_DLGAP4 were assessed. miR-143 was negatively regulated by circ_DLGAP4 in MCs (Fig. 3C,D). miR-143 expression was obviously decreased in T2D patients with DKD, DKD patients with microalbuminuria, and DKD patients with high eGFR compared to the corresponding controls, as exhibited in Fig. 3E–G. Consistently, miR-143 was also downregulated in rat DKD model (Fig. 3H). Next, circ_DLGAP4 and miR-143 in cell lysis were greatly pulled down and enriched by circ_DLGAP4 specific probe in MCs in Fig. 3I, J. In Fig. 3K, biotin-coupled miR-143 captured a fold change of circ_DLGAP4 in the complex. Afterward, RNA immunoprecipitation (RIP) was performed using Ago2 antibody in MCs cells transfected with miR-143 mimic or mimic NC. We proved that the enrichment of circ_DLGAP4 was increased by miR-143 mimic in Fig. 3L. The direct binding sites between circ_DLGAP4 and miR-143 were presented in Fig. 3M. The luciferase reporter activity of Wt-circ_DLGAP4 in MCs cells co-transfected with miR-143 mimic was reduced, while induced by the inhibitor in Fig. 3N.

### miR-143 suppressed the proliferation and fibrosis of MCs by targeting ERBB3

**Fig. 4:**
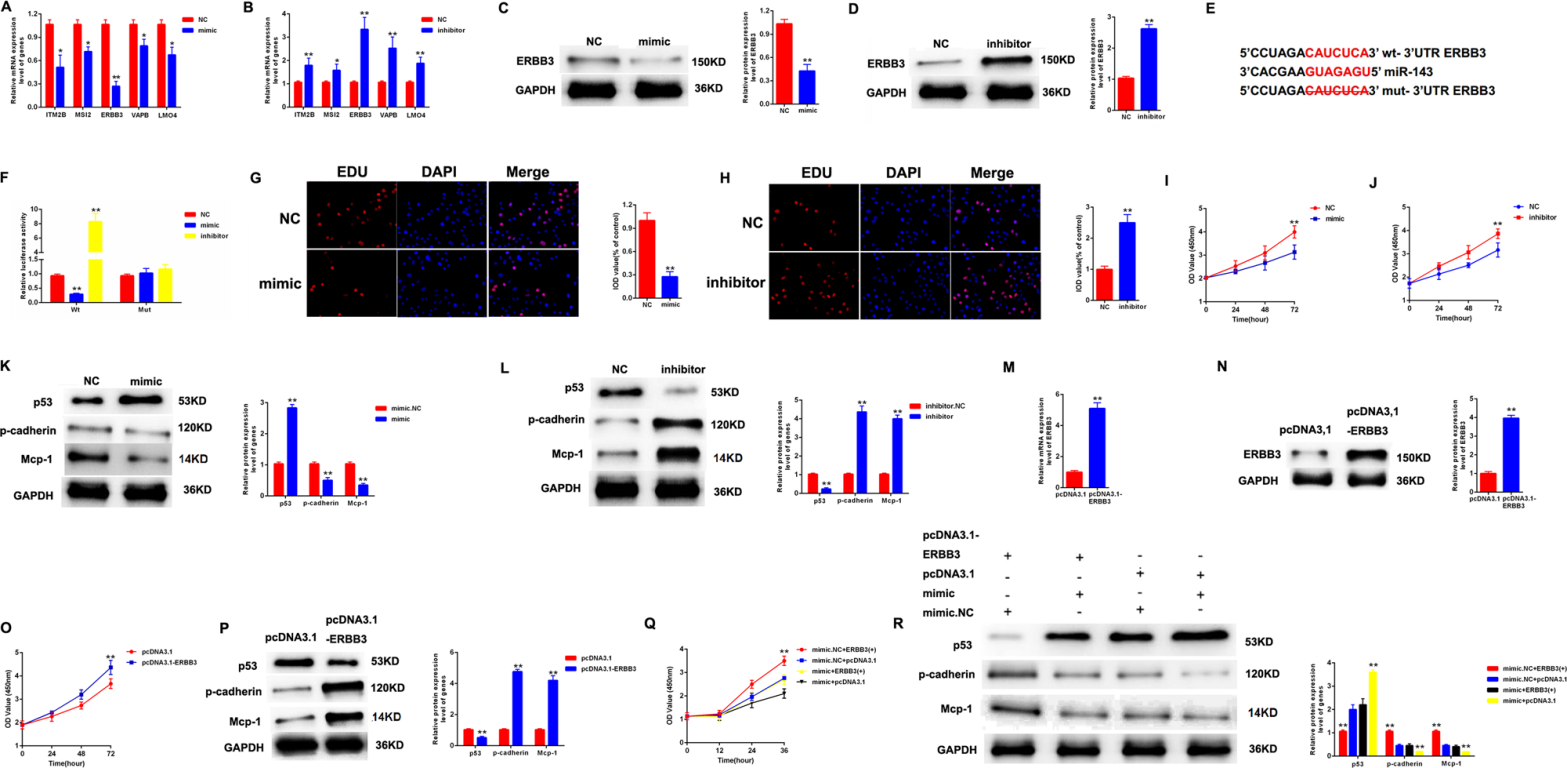
miR-143 suppressed proliferation and fibrosis of MCs by targeting ERBB3. **A** Five genes were screened in MCs transfected with mimic-miR-143. **B** Five genes were screened in MCs transfected with inhibitor-miR-143. **C**, **D** The protein expression level of ERBB3 in MCs transfected by mimic-miR-143 or inhibitor-miR-143. **E** The direct binding sites between miR-143 and ERBB3 were presented. **F** Luciferase reporter assay was performed to confirm the direct binding relationship between miR-143 and ERBB3. **G**, **H** Proliferating MCs were labeled with EdU after transfection with mimic-miR-143 or inhibitor-miR-143. **I**, **J** Proliferating MCs were labeled with CCK-8 after transfection with mimic-miR-143 or inhibitor-miR-143. **K**, **L** The protein expression level of p-53, p-cadherin, and Mcp-1 in MCs transfected with mimic-miR-143 or inhibitor-miR-143. **M**, **N** Efficiency of pcDNA3.1-ERBB3 were confirmed by qPCR and western blotting. MCs were transfected with pcDKDA3.1-ERBB3 or vector. **O** CCK-8 assay was carried out to evaluate cell proliferation. MCs were transfected with pcDNA3.1-ERBB3 or vector. **P** p-53, p-cadherin, and Mcp-1 protein expression in MCs transfected with pcDNA3.1-ERBB3 or vector. **Q** The growth of MCs was detected after transfected by mimic-miR-143 or pcDNA3.1-ERBB3. **R** p-53, p-cadherin, and Mcp-1 protein expression in MCs transfected by mimic-miR-143 or pcDNA3.1-ERBB3. Three independent experiments were conducted. Error bars stand for the mean ± SD of at least triplicate experiments; *P < 0.05, **P < 0.01.

Next, five genes were screened in MCs transfected with mimic-miR-143 and inhibitor-miR-143. In Fig. 4A, B, ERBB3 mRNA expression exhibited the most obvious change after the treatment of mimic-miR-143 and inhibitor-miR-143. Then, in Fig. 4C, D, the protein expression of ERBB3 in MCs was negatively modulated by miR-143. In Fig. 4E, luciferase reporter vectors containing Wt-ERBB3 or Mut-NOTCH2 were constructed. In Fig. 4F, mimic of miR-143 reduced the luciferase activity of Wt-ERBB3, while inhibitor of miR-143 induced the luciferase activity in MCs. In addition, proliferating MCs were labeled with EdU and CCK-8 after transfection with mimic-miR-143 or inhibitor-miR-143. mimic-miR-143 greatly decreased MCs proliferation, whereas inhibitor-miR-143 increased cell proliferation in Fig. 4G–J. Next, we observed the protein expression level of p-53, p-cadherin, and Mcp-1 in MCs was downregulated by mimic-miR-143, while upregulated by inhibitor-miR-143, as shown in Fig. 4K, L. In Fig. 4M, N, efficiency of pcDKDA3.1-ERBB3 were confirmed by qPCR and western blotting. Cell proliferation and fibrosis were greatly triggered by ERBB3 overexpression in Fig. 4O, P. Moreover, MCs were transfected by mimic-miR-143 or pcDKDA3.1-ERBB3. As indicated in Figs. 4Q, R, the proliferation and fibrosis of MCs was inhibited by mimic-miR-143, which was reversed by the increase of ERBB3.

### circ_DLGAP4 promoted proliferation and fibrosis of MCs by regulating ERBB3/NF-κB/MMP-2 axis and sponging miR-143

**Fig. 5:**
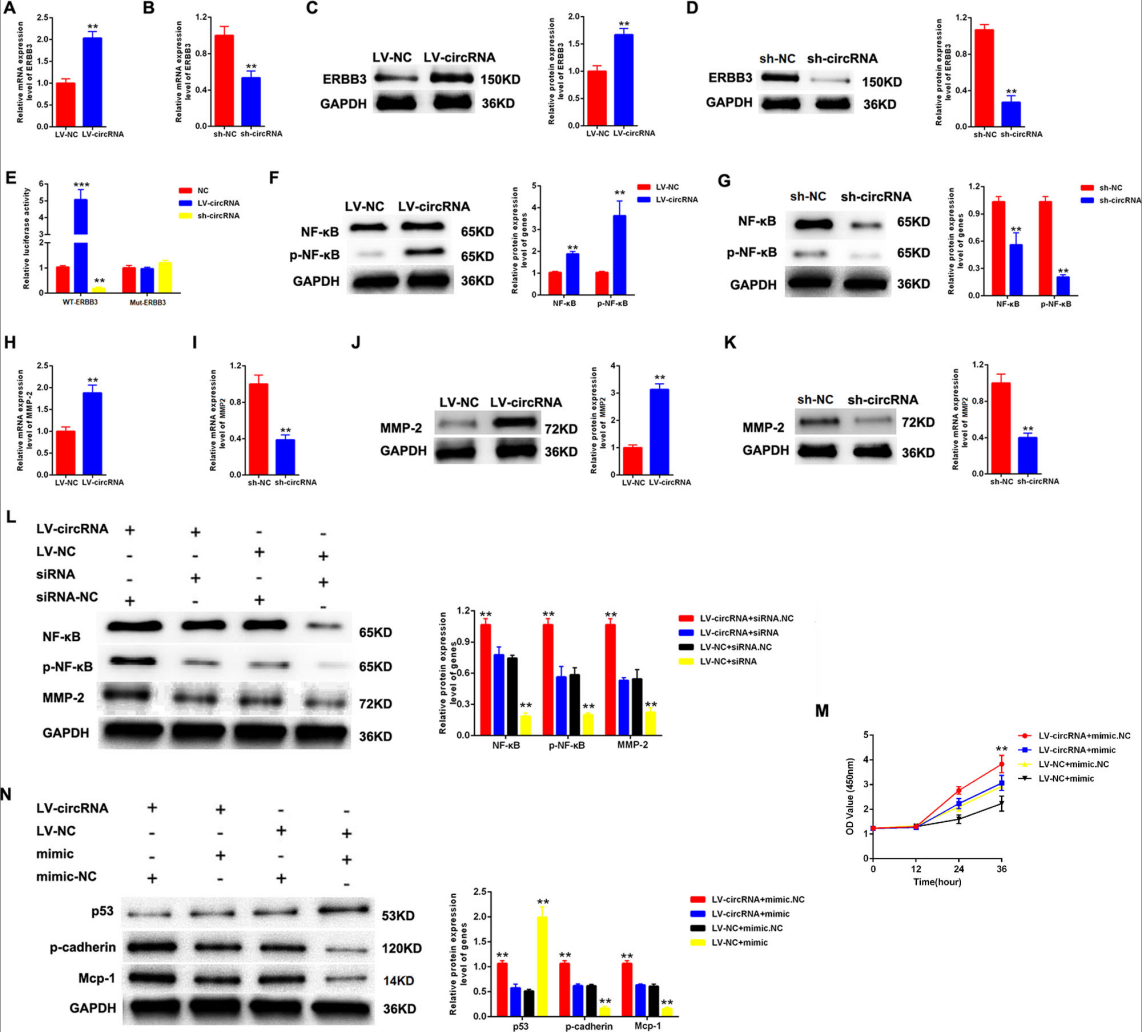
circ_DLGAP4 promoted proliferation and fibrosis of MCs by regulating ERBB3/p-NF-κB/MMP-2 axis and sponging miR-143. **A**, **B** The mRNA expression level of ERBB3 in MCs infected with LV-circ_DLGAP4 or sh-circ_DLGAP4. **C**, **D** The protein expression level of ERBB3 in MCs infected with LV-circ_DLGAP4 or sh-circ_DLGAP4. **E** The mutant-type reporter gene (Mut-DLGAP4 reporter) and wild-type reporter gene (Wt-DLGAP4 reporter) were established. MCs were co-transfected with LV-circ_DLGAP4 or sh-circ_DLGAP4 with Mut-DLGAP4 reporter or Wt-DLGAP4 reporter. **F**, **G** The expression level of NF-κB/p-NF-κB was measured by western blotting assay. MCs were transfected by LV-circ_DLGAP4 or sh-circ_DLGAP4. **H**, **I** The mRNA expression level of MMP-2 in MCs. **J**, **K** The protein expression level of MMP-2 in MCs. **L** The expression level of p-NF-κB and MMP-2 in MCs treated with LV-circ_DLGAP4 or ERBB3 siRNA. **M** Proliferation of MCs transfected with LV-circ_DLGAP4 or mimic-miR-143. **N** The protein expression level of p-53, p-cadherin, and Mcp-1 in MCs treated with LV-circ_DLGAP4 or mimic-miR-143. Three independent experiments were conducted. Error bars stand for the mean ± SD of at least triplicate experiments; **P < 0.01, ***P < 0.001.

Then, we evaluated the correlation between circ_DLGAP4 and ERBB3. Firstly, we found that the expression level of ERBB3 was positively regulated by circ_DLGAP4 in MCs, as displayed in Fig. 5A–D. In Fig. 5E, the Mut-DLGAP4 reporter and Wt-DLGAP4 reporter were established. MCs were co-transfected with LV-circ_DLGAP4 or sh-circ_DLGAP4 with Mut-DLGAP4 reporter or Wt-DLGAP4 reporter. It was evidenced that the luciferase activity of Wt-DLGAP4 reporter was induced by LV-circ_DLGAP4, while reduced by LV-circ_DLGAP4. In Fig. 5F, the expression level of NF-κB was induced by LV-circ_DLGAP4 and p-NF-κB was enhanced by LV-circ_DLGAP4. In Fig. 5G, an opposite result was exhibited in sh-circ_DLGAP4 group. For another, MMP-2 expression was also positively modulated by circ_DLGAP4 in MCs, as indicated in Fig. 5H–K. Then, MCs were treated with LV-circ_DLGAP4 or ERBB3 siRNA. The protein expression level of p-NF-κB and MMP-2 was induced by LV-circ_DLGAP4, which could be reduced by ERBB3 siRNA in Fig. 5L. In Fig. 5M, N, proliferation and fibrosis of MCs was increased by LV-circ_DLGAP4, which could be decreased by mimic-miR-143.

### The effects of exo-circ_DLGAP4 on miR-143 and ERBB3/NF-κB/MMP-2 axis in MCs cells

**Fig. 6:**
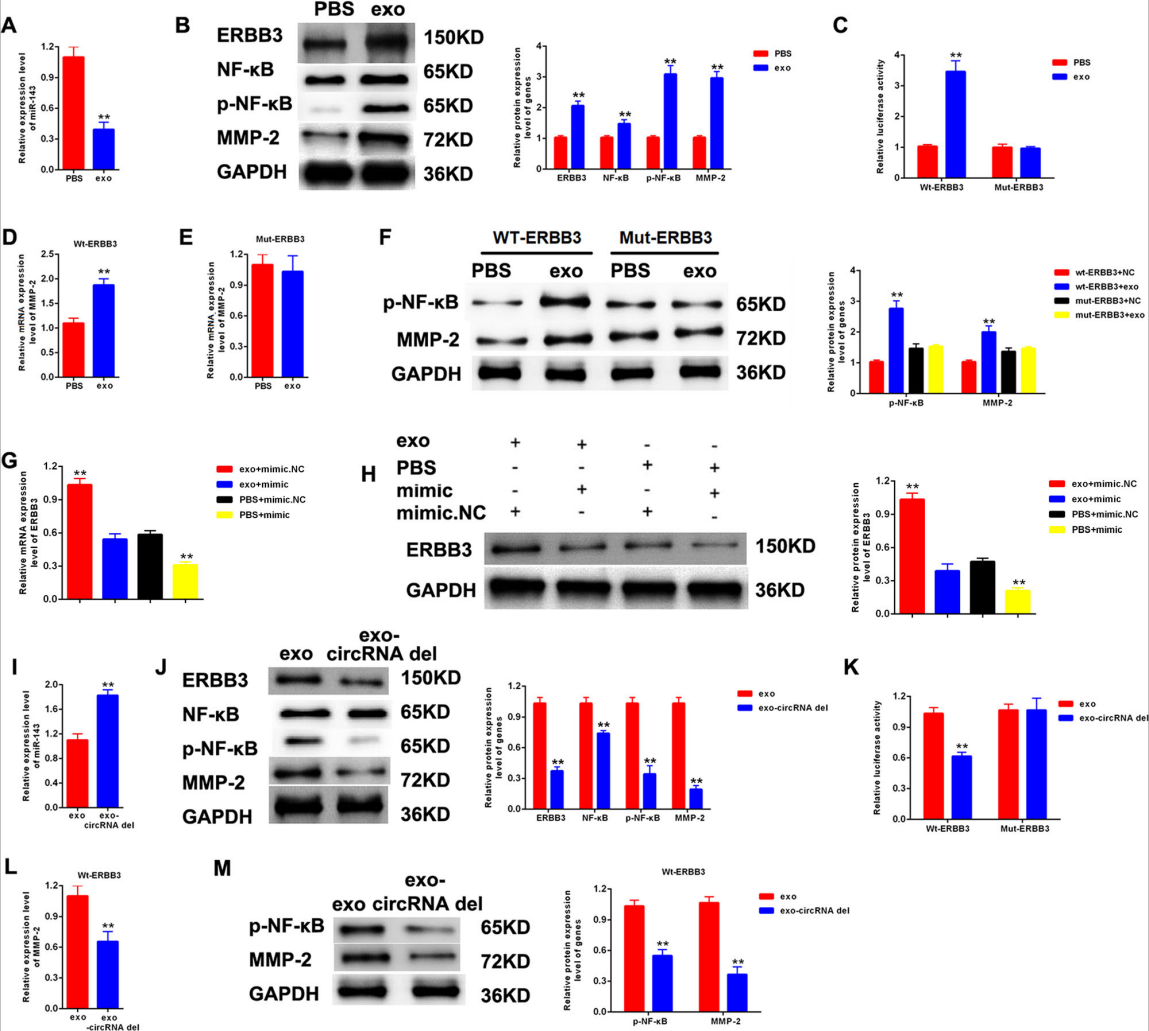
The effects of exo-circ_DLGAP4 on miR-143 and ERBB3/p-NF-κB/MMP-2 axis in MCs. **A** The expression level of miR-143 was detected in MCs treated with NC or exosomes. **B** The protein expression level of ERBB3/p-NF-κB/MMP-2 axis was detected in MCs treated with NC or exosomes. **C** The mutant-type reporter gene (Mut-ERBB3 reporter) and wild-type reporter gene (Wt-ERBB3 reporter) were established. MCs were co-transfected with NC or exosome with Mut-ERBB3 reporter or Wt-ERBB3 reporter. Luciferase reporter assay was performed to confirm the relationship between exosomes and ERBB3. **D**–**F** The protein expression level of p-NF-κB and MMP-2 was detected in Wt-ERBB3 group and Mut-ERBB3 group. **G**, **H** The protein expression level of ERBB3 in MCs treated with exosomes or mimic-miR-143. **I** The expression level of miR-143 was detected in MCs treated with exosomes or exo-circRNA del. **J** The protein expression level of ERBB3/p-NF-κB/MMP-2 axis was detected in MCs treated with exosomes or exo-circRNA del. **K** The Mut-ERBB3 and Mt- ERBB3 reporter were established. MCs transfected with Mut-ERBB3 reporter or Wt-ERBB3 reporter were treated by exosomes or exo-circRNA del. Luciferase reporter assay was performed to confirm the relationship between exo-circRNA del and ERBB3. **L** The mRNA expression level of MMP-2 was detected in Wt-ERBB3 group. **M** The protein expression level of MMP-2 and p-NF-κB was detected in Wt-ERBB3 group. Three independent experiments were conducted. Error bars stand for the mean ± SD of at least triplicate experiments; **P < 0.01.

Next, we assessed the influence of exo-circ_DLGAP4 on miR-143 and ERBB3/NF-κB/MMP-2 expression in MCs cells. miR-143 was reduced while ERBB3/p-NF-κB/MMP-2 protein expression was induced by exosomes in MCs, as shown in Fig. 6A, B. For another, the luciferase reporter activity of Wt-ERBB3 was activated by exosomes in MCs in Fig. 6C. In Fig. 6D–F, the protein expression level of p-NF-κB and MMP-2 was upregulated in Wt-ERBB3 group, while no change was observed in Mut-ERBB3 group. In Fig. 6G, H, the protein expression level of ERBB3 in MCs was upregulated by exosomes, which was downregulated by mimic-miR-143. Then, expression level of miR-143 in MCs treated with exo-circRNA was significantly induced (Fig. 6I). Meanwhile, we detected the protein expression level of ERBB3/p-NF-κB/MMP-2 in MCs treated with exosomes or exo-circRNA del. We observed that ERBB3/p-NF-κB/MMP-2 protein expression was obviously restrained in exo-circRNA del group in Fig. 6J. The luciferase reporter activity of Wt-ERBB3 was markedly inactivated by exo-circRNA del in MCs, as manifested in Fig. 6K. Subsequently, it was shown that the expression level of MMP-2 and p-NF-κB was depressed by exo-circRNA del under Wt-ERBB3 treatment in Fig. 6L, M.

### Overexpression of circ_DLGAP4 promoted DKD progression in vivo

**Fig. 7:**
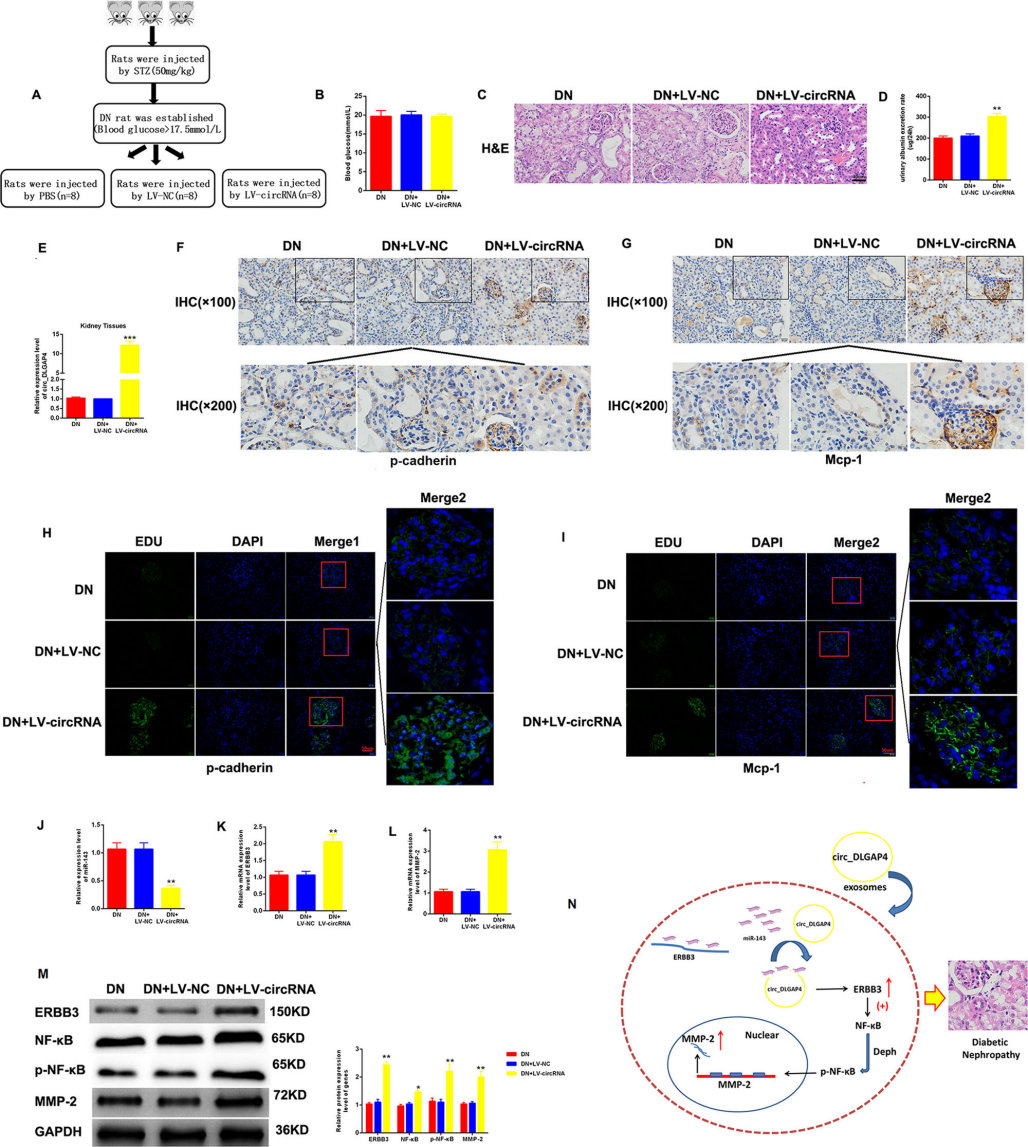
Overexpression of circ_DLGAP4 promoted DKD progression in vivo. **A** Graphical representation of DKD model treated with PBS, LV-NC, or LV-circ_DLGAP4. **B** Blood glucose was detected in DKD + PBS group, DKD + LV-NC group, and DKD + LV-circ_DLGAP4 group. **C** HE staining results of the DKD + PBS group, DKD + LV-NC group, and DKD + LV-circ_DLGAP4 group. **D** Urinary albumin excretion rate was measured in the DKD PBS group, DKD + LV-NC group, and DKD + LV-circ_DLGAP4 group. **E** circ_DLGAP4 expression in kidney tissues from DKD + PBS group, DKD + LV-NC group, and DKD + LV-circ_DLGAP4 group. **F**, **G** The expression level of p-cadherin and Mcp-1 was measured by IHC in DKD + PBS group, DKD + LV-NC group, and DKD + LV-circ_DLGAP4 group. **H**, **I** The expression level of p-cadherin and Mcp-1 was assessed using IF assays in in DKD + PBS group, DKD + LV-NC group, and DKD + LV-circ_DLGAP4 group. **J**–**L** The expression level of miR-143, ERBB3, and MMP-2 mRNA were measured in DKD + PBS group, DKD + LV-NC group, and DKD + LV-circ_DLGAP4 group. **M** The protein expression levels of ERBB3/p-NF-κB/MMP-2 axis was detected in DKD + PBS group, DKD + LV-NC group, and DKD + LV-circ_DLGAP4 group. **N** Schematic diagram of mechanism of circ_DLGAP4/miR-143/ERBB3/p-NF-κB/MMP-2 in DKD progression was provided. Three independent experiments were conducted. Error bars stand for the mean ± SD of at least triplicate experiments; **P < 0.01.

Then, in vivo assays were carried out to assess the effect of circ_DLGAP4 on DKD progression. As displayed in Fig. 7A, graphical representation of DKD model treated with PBS, LV-NC, or LV-circ_DLGAP4. Blood glucose in the DKD + PBS group, DKD + LV-NC group, and DKD + LV-circ_DLGAP4 group exhibited no significant difference in Fig. 7B. HE staining in Fig. 7C indicated that LV-circ_DLGAP4 group exhibited great glomerular shrinkage and fibrosis. Glomerular mesangial proliferation was enhanced in DKD + LV-circ_DLGAP4 group than DKD + PBS group or DKD + LV-NC group. In addition, urinary albumin excretion rate was increased in DKD + LV-circ_DLGAP4 group, as indicated in Fig. 7D. Circ_DLGAP4 expression was exhibited in kidney tissues from DKD + PBS group, DKD + LV-NC group, and DKD + LV-circ_DLGAP4 group in Fig. 7E. Then, the expression level of p-cadherin and Mcp-1 was significant induced in LV-circ_DLGAP4 group, as proved by IHC and IF assay in Fig. 7F–I. miR-143 level was repressed by circ_DLGAP4, whereas ERBB3 and MMP-2 mRNA levels were increased by circ_DLGAP4 in Fig. 7J–L. Meanwhile, the protein expression of ERBB3/p-NF-κB/MMP-2 was obviously triggered in LV-circ_DLGAP4 group (Fig. 7M). Finally, a schematic diagram of mechanism of circ_DLGAP4/miR-143/ERBB3/NF-κB/MMP-2 in DKD progression was provided in Fig. 7N.

## Discussion

In this research, we discovered that circ_DLGAP4 was increased in the exosomes isolated from HG-treated MCs, DKD patients, and DKD rat models. Exo-circ_DLGAP4 greatly promoted proliferation and fibrosis of MCs cells. miR-143 was predicted as the target of circ_DLGAP4. We verified the direct association between miR-143 and circ_DLGAP4. Then, ERBB3 was predicted as the downstream target for miR-143. Circ_DLGAP4 could promote proliferation and fibrosis of MCs by sponging miR-143 and regulating ERBB3/NF-κB/MMP-2 axis.

Glomerular MCs are a crucial member of renal glomerulus, and they can play a major role in physiological functions and in pathogenesis of kidney diseases^22^. MCs are able to keep the structural architecture of the glomerular capillary similar to microvascular pericytes function. Meanwhile, they contribute to the homeostasis of mesangial matrix, modulate filtration surface area, and phagocytose apoptotic cells. Hypertrophy and proliferation of MCs, as well as fibrosis have been identified as common biological responses of cell injury, leading to DKD progression.

It has been elucidated that circ-DLGAP4 expression is decreased in AIS patients^23^. Circ-DLGAP4 is reduced and negatively correlates with miR-143 expression in AIS patients. In addition, circ-DLGAP4 can inhibit ischemic stroke outcomes via sponging miR-143 to modulate blood–brain barrier integrity^20^. Meanwhile, epigenetic remodeling and DLGAP4 dysregulation is closely linked with early-onset cerebellar ataxia^24^. However, there is still no study, which can identify exo-circ-DLGAP4 as a biomarker for DKD. therefore, our study evaluated the detailed function of exo-circ-DLGAP4 expression in DKD. We successfully isolated exosomes from culture media of MCs and human serum samples. Circ_DLGAP4 in exosomes of HG-incubated MCs, DKD patients, and DKD rat models was increased. Exo-circ_DLGAP4 promoted growth and fibrosis of MCs cells, while loss of circ_DLGAP4 in exosomes was able to repress DKD progression.

Then, miR-143 was predicted as a target of circ_DLGAP4 using bioinformatics method. miR-143 is involved in many diseases. miR-143 can depress colorectal tumorigenesis via targeting KRAS^25^. miR-143 plays an important role in mitochondrial function and glucose metabolism in diabetes mellitus^26^. Elevated miR-143 has been shown in saphenous vein smooth muscle cells from T2D patients^27^. In addition, loss of miR-143/145 cluster results in hydronephrosis in mice^28^. Overexpression of miR-143 contributes to glomerular filtration barrier impairment by targeting proteoglycans^29^. miR-143 was confirmed as the direct target of circ_DLGAP4. miR-143 could repress MCs proliferation and fibrosis, which was reversed by circ_DLGAP4.

ERBB protein family members are type 1 tyrosine kinase receptors and they contains four receptor tyrosine kinases related to the EGFR^30^. This family includes ERBB1, ERBB2, ERBB3, and ERBB4 and it participates in various cellular processes, including cell proliferation, migration, and metabolism^31,32^. The activated ERBB proteins can recruit signaling complexes, including MAPK, PI3K/Akt, STATs, NF-κB, and mTOR signaling^33^. ERBB3 was predicted as a target for miR-143. Circ_DLGAP4 induced diabetic nephropathy progression by activating ERBB3.

There is increasing evidence showing that DKD is related to mesangial inflammation^34^. The activation of NF-κB might exert a significant role in renal injury via inducing inflammatory factors in diabetes^35^. Thus, inhibition of the NF-κB signaling may indicate a new therapeutic target for DKD. Here, in our study, we found that the loss of circ_DLGAP4 restrained NF-κB signaling via sponging miR-143 and targeting ERBB3.

For another, upregulated synthesis or downregulated degradation of type IV collagen by MCs can lead to ECM accumulation, which can lead to mesangial lesion expansion^36^. Matrix metalloprotease-2 (MMP-2) is a type IV collagenase and it can efficiently cleave collagen IV. As reported, MMP-2 in experimental and human DKD has been identified^37^. In addition, MMP-2 is closely associated with glomerulonephritis^38^. We found that the protein expression of MMP-2 in MCs was upregulated by exosomes- circ_DLGAP4, which was downregulated by mimic-miR-143.

In conclusion, this was a first study revealing the value of exo-circ_DLGAP4 circ-DLGAP4 for DKD. Its correlation with miR-143 and ERBB3/NF-κB/MMP-2 in DKD, which provided novel insight on the value of circRNAs in DKD. These data encouraged a future investigation of the potential of exo-circ_DLGAP4 for DKD.

## Materials and methods

### Subjects

Seventy-eight T2D patients were enrolled in our research. T2D diagnosis was based on the American Diabetes Association’s criteria. Meanwhile, patients were assigned in two groups, one with 34 subjects that were without DKD, and the other with 38 patients, with DKD confirmed with urine albumin/creatinine ratio >30 mg/g. Meanwhile, patients with albumin/creatinine values of 30–299 mg/g or ≥300 mg/g were considered microalbuminuria or macroalbuminuria, respectively. The study was carried out under the approval by the Ethics Committee of Qingpu Branch of Zhongshan Hospital Affiliated to Fudan University. Participants all provided their informed written consent.

### Experimental animals

Forty healthy and SPF male SD rats (weighed 180 ± 20 g) were obtained from Beijing HFK Bioscience Co. Ltd. (Beijing, China). Rats were maintained in a clean environment with a temperature of 20–25 °C. DKD rats were induced via injecting 55 mg/kg streptozotocin in the tail vein, while NC rats were injected using an equal volume of solvent. Rats were divided into a NC group, DKD group, DKD + PBS group, DKD + LV-NC group, and DKD + LV-circ_DLGAP4 group. *n* = 8 in each group. LV-circ_DLGAP4 or LV‐NC (120 µL) was injected into DKD rats via tail vein. At the end of the third week after injection, all rats were anesthetized using 50 mg/kg intraperitoneal sodium pentobarbital and sacrificed through drawing-out blood from their hearts. Kidneys tissues of the rats were harvested. One was fixed using 4% paraformaldehyde and the other one was frozen in liquid nitrogen. This study was performed under the guidelines of the National Health and Medical Research Council of China’s Code for the care and use of animals.

### Cell culture

The rat MCs were purchased from Academy of Sciences of Shanghai (Shanghai, China). MCs were maintained at 37 °C in DMEM (Hyclone, UT, USA) with 10% FBS (Gibco, CA, USA) in an atmosphere containing 5% CO_2_. Cells were treated with the NG (5.5 mM glucose) or HG (25 mM glucose).

### Cell transfection

MCs were transfected with miR-143 mimics, miR-143 inhibitors, LV-circ_DLGAP4, sh-circ_DLGAP4, ERBB3 siRNA, pcDKDA3.1-ERBB3, or the corresponding NC (RiboBio Corporation, Guangzhou, China) using Lipofectamine 3000 (Invitrogen, Carlsbad, CA, USA). The medium was replaced using fresh culture medium after transfection for 4–6 h.

### Isolation of exosomes

Exosomes in cultural medium and serum were isolated using differential centrifugation at 4 °C. After removing cells and other debris via 300 and 3000 × *g* centrifugation, the supernatant was centrifuged at 10,000 × *g* for half an hour. In the end, the supernatant was centrifuged at 110,000 × *g* for 70 min at 4 °C. We obtained exosomes from the pellet and resuspended them in PBS.

### Transmission electron microscopy assay

Exosome pellet was maintained in 2.5% glutaraldehyde at pH 7.2 and fixed for a whole night. Samples were rinsed using PBS buffer and postfixed using 1% osmium tetroxide for 1 h. The samples were embedded in 10% gelatin and fixed using glutaraldehyde at 4 °C. The samples were dehydrated for 10 min in various concentrations of alcohol. Then, pure alcohol was exchanged by propylene oxide, and specimens were infiltrated by various concentrations of Quetol-812 epoxy resin mixed with propylene oxide for 3 h. Samples were embedded in pure, fresh Quetol-812 epoxy resin and then polymerized. Then, ultrathin sections were cut using a Leica UC6 ultramicrotome and post-stained with uranyl acetate for 10 min and with lead citrate for 5 min, and then we observed using a FEI Tecnai T20 transmission electron microscope at 120 kV.

### CCK-8 assay

CCK-8 assay (Dojindo, Kumamoto, Japan) was carried out to evaluate the proliferation of MCs. Cells were grown into 96-wells plates. After indicated treatment, we washed the cells using PBS, and CCK-8 reagent was added to the well for 2 h. The optical density value of absorbance at 450 nm was tested by a microplate reader (BioRad, Richmond, CA, USA).

### EdU assay

EdU assay was performed using Cell-Light EdU Apollo 567 In Vitro Imaging Kit (Ribobio, Guangzhou, China). Briefly, cells were seeded into 96-well plates. A total of 50 μM EdU was used to incubate the cells for 2 h at 37 °C. Cells were fixed with 4% paraformaldehyde then stained by Hoechst 33342 and Apollp reaction cocktail. The images were captured under a fluorescence microscopy (Nikon, Melville, NY, US).

### Cell cycle analysis

Cells were collected using trypsinization and fixed by 70% ethanol at −20 °C for a whole night. Then, cells were treated with 50 μL of 100 μg/mL boiled RNase for half an hour followed by staining with 200 μL propidium iodide. A flow cytometry was utilized to acquire cell cycle and data were analyzed using the ModFit LT software (Verity Software House Inc, Topsham, ME, USA).

### qRT-PCR

Total RNA was obtained using TRIzol reagent. Reverse-transcribed cDNA was manufactured using a Prime-script RT reagent Kit based on manufacturer’s instruction (TaKaRa, Dalian, China). RT-PCR was conducted using a SYBR Premix Ex Taq (TaKaRa, Dalian, China) on an Applied Biosystems StepOne-Plus real-time PCR system. Primers were obtained from GeneCopoeia (Guangzhou, Guangdong, China) and exhibited in Table 1. Subsequently, relative expression of genes was calculated by 2^−ΔΔCT^.

**Table 1:** Primers used for real-time PCR.

Genes	Forward (5′–3′)	Reverse (5′–3′)
GAPDH	GGAGCGAGATCCCTCCAAAAT	GGCTGTTGTCATACTTCTCATGG
ERBB3	TTCCGAGATGGGCAACTCTC	CTTGCAGACTTCGTGACAGG
MMP-2	TACAGGATCATTGGCTACACACC	GGTCACATCGCTCCAGACT
U6 miR-143	CTCGCTTCGGCAGCACA GCGGCGGTGAGATGAAGCACTG	GGTTCACAGGCACATTCGTA ATCCAGTGCAGGGTCCGAGG

### Western blot

Briefly, cells and tissues were lysed, and protein concentration of total cell lysates was tested by using the BCA assay (Beyotime, Shanghai, China). About 25 μg whole cell lysates were loaded to electrophoresis and then transferred to PVDF membranes (Merck Millipore, USA). After blocked in 5% nonfat milk for 1 h, the membranes were incubated primary antibodies: anti-CD63 (cat. no. ab134045; Abcam, Cambridge, UK, 1:1000), anti-Alix (cat. no. ab186728; Abcam, Cambridge, UK, 1:1000), anti-Tsg101 (cat. no. ab125011; Abcam, Cambridge, UK, 1:1000), anti-p-cadherin (cat. no. ab51034; Abcam, Cambridge, UK, 1:1000), anti-Mcp-1 (cat. no. ab25124; Abcam, Cambridge, UK, 1:1000), anti-GAPDH (cat. no. ab9484; Abcam, Cambridge, UK, 1:1000), anti-p-53 (cat. no. ab26; Abcam, Cambridge, UK, 1:1000), anti-ERBB3 (cat. no. ab32121; Abcam, Cambridge, UK, 1:1000), anti-NF-κB (cat. no. ab220803; Abcam, Cambridge, UK, 1:1000), anti-p-NF-κB (cat. no. ab222494; Abcam, Cambridge, UK, 1:1000), and anti-MMP-2 (cat. no. ab92536; Abcam, Cambridge, UK, 1:1000) for overnight at 4 °C. After incubated with corresponding secondary antibodies goat anti-rabbit IgG (cat. no. ab205718; Abcam, Cambridge, UK, 1:5000) and goat anti-mouse IgG (cat. no. ab6789; Abcam, Cambridge, UK, 1:5000), the membrane was washed using TBST, and then incubated with BeyoECL Plus chemical luminescence solution (Beyotime, Shanghai, China). Finally, the membrane was imaged by a ChemiDoc XRS imaging system and analyzed by the QuantityOne software (BioRad, Hercules, CA, USA).

### Immunofluorescent staining

Dissected tissues were frozen in liquid nitrogen‐cooled isopentane and embedded in OCT medium. To carry out immunofluorescent staining, serial cross sections were cut on a cryostat at −20 °C. Afterward, the sections were fixed using cold acetone for 10 min and incubated in a 1% BSA/PBS solution. The sections were incubated with antibody cocktail of primary antibodies against Mcp-1 and p-cadherin (1:100, Santa Cruz, Dallas, TX, USA) overnight at 4 °C. Next day, the sections were incubated with secondary antibody (1:500, Santa Cruz, Dallas, TX, USA) for 1 h. The sections were mounted in Mowiol mounting medium (Merck KGaA, Darmstadt, Germany). We visualized the slides using an Axio Observer Z1 microscope (Carl Zeiss, Jena, Germany) by conventional wide‐field fluorescence microscopy.

### Luciferase activity assay

The putative sequences of the binding site in circ_DLGAP4/ERBB3 and the mutated sequences were cloned into a pmirGlO Dual‐luciferase Vector (Promega, Madison, WI, USA). The report vector Wt-circ_DLGAP4/ERBB3 or Mut- circ_DLGAP4/ERBB3 was co‐transfected with inhibitor-miR-143, mimic- miR-143, LV-circ_DLGAP4, or sh-circ_DLGAP into MCs for luciferase assay, using a Dual‐Luciferase Reporter Assay System (Promega, Madison, WI, USA).

### RNA immunoprecipitation

RIP assay was conducted using Magna RIP RNA Binding Protein Immunoprecipitation Kit (Millipore, Billerica, MA, USA). Cells were lysed using RIP lysis buffer. Whole cell extract was incubated with RIPA buffer with magnetic beads conjugated with human Ago2 antibody (Millipore, Billerica, CA, USA) for 8 h. Meanwhile, normal mouse IgG (Millipore, Billerica, CA, USA) served as a negative control. Then, immunoprecipitated RNA was extracted and subjected to qRT-PCR analysis.

### Biotinylated RNA pull-down assay

For circ_DLGAP4 pulled down miRNAs, the biotinylated-circ_DLGAP4 probe was incubated with C-1 magnetic beads and then incubated with cells at 4 °C for a whole night, followed by eluted and qRT-PCR. For miR-143 pulled down circ_DLGAP4, cells with circHIPK3 overexpression were transfected with biotinylated miR-143 mimics or mutant. After harvested, lysed, and sonicated, cells were incubated with C-1 magnetic beads and then subjected to qRT-PCR.

### Immunohistochemical staining

For immunohistochemical staining analysis, before performing antigen retrieval, tissue sections were dewaxed and rehydrated. The slides were incubated with anti-p-cadherin and Mcp-1 (1:100, Santa Cruz, Dallas, TX, USA) overnight, and incubated with an HRP-conjugated secondary antibody for 1 h. Then, DAB was utilized for color development and dark brown staining was considered to be positive.

### Histopathology examination

The histopathology examination was carried out with HE staining. Samples were put in 10% formaldehyde solution, dehydrated in ethanol gradient, embedded in paraffin, and cut down into slices of 4 μm. After deparaffinage, the samples were stained by HE. Slices were mounted and observed using a light microscope.

### Statistical analysis

Statistics was analyzed by using SPSS 22.0 software (SPSS, Chicago, IL, USA). Student’s *t* test (two groups) and one-way ANOVA (no less than three groups) were utilized to analyze the significance. A probability value *P* < 0.05 was considered to be statistically significant.

## Supplementary information

Supplement Table 1

## Data Availability

All data are available upon request.
